# Choline diet improves serum lipid parameters and alters egg composition in breeder ducks

**DOI:** 10.1002/vms3.798

**Published:** 2022-04-05

**Authors:** Jianlou Song, Xuefeng Shi, Xingzheng Li, Jiangxia Zheng

**Affiliations:** ^1^ Key Laboratory of Animal Genetics, Breeding and Reproduction of the Ministry of Agriculture, College of Animal Science and Technology, National Engineering Laboratory for Animal Breeding China Agricultural University Beijing China

**Keywords:** atherosclerosis index, breeder duck, cholesterol, choline, lipid parameter

## Abstract

**Background:**

Choline is an important nutrient, playing key roles in numerous metabolic pathways relevant to animal health.

**Objectives:**

The objective of this study was to evaluate the effect of dietary choline on the lipid parameters, cardiovascular health (CVH), and levels of egg trimethylamine (TMA) and cholesterol in breeder ducks during the late laying period.

**Methods:**

A total of 60 Jingjiang ducks were randomly separated into six replicates of 10 ducks each. After peak production until 65 weeks of age, the birds were fed a control basal diet. The same ducks served as the control group until 65 weeks of age, when the same ducks served as the choline‐supplemented group, after 15 days of dietary choline supplementation at 2955 mg/kg choline above and over the basal diet initially provided. The 15 days of choline supplementation included an initial 5‐day acclimatisation period.

**Results:**

Dietary choline supplementation increased serum TMA (*p* < 0.01), high‐density lipoprotein cholesterol, very low‐density lipoprotein, and triglyceride levels in older breeder ducks. However, it did not change the levels of trimethylamine N‐oxide but decreased the atherosclerosis index compared with those of the control group (*p* < 0.01). Moreover, it increased (*p* < 0.01) the egg yolk TMA levels but did not change the concentrations of cholesterol in egg yolk.

**Conclusions:**

Dietary choline supplementation had a beneficial effect on lipid parameters and CVH in older breeder ducks, although it increased the serum and egg yolk TMA levels.

## Graphical abstract

Dietary choline supplementation had a beneficial effect on lipid parameters and cardiovascular health of older breeder ducks, as it decreased atherosclerosis index, a predictor of cardiovascular disease risk. Furthermore, although it increased the egg yolk trimethylamine levels in ducks, it did not alter the egg yolk cholesterol concentration.

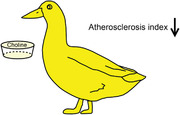

## 1 INTRODUCTION

Duck production is an important aspect of the poultry industry in China. The rearing of breeder ducks in cage‐housing settings is becoming more widespread because this system can avoid contamination of hatching eggs, improve laying performance and feed conversion rates, and increase flock density; these characteristics are convenient for intensive duck production units (Zhang, 2014). In general, the laying rate of breeder ducks reaches over 90% beginning 23 weeks of age, and peak production continues until about 53 weeks of age with reasonable management. However, after the peak of egg‐laying, the restricted activity of the caged breeder ducks leads to fat deposition. Especially in the winter, it is difficult to optimise the diet of breeder ducks during the late laying period. This fact highly increases the risk of obesity among the breeder ducks, which further affects the quality of their performance (in terms of laying rate, eggshell quality and nutrients in eggs) (Zheng, 2014). Obesity is associated with changes in serum lipid parameters, which are also a major cause of cardiovascular disease (CVD) in ducks and other animals (Brown & Hazen, 2014). Therefore, it is necessary to determine lipid parameters and improve the health of breeder ducks during the late laying period.

Choline is an important nutrient. It has been reported to play key roles in numerous metabolic pathways relevant to animal health (Kasper et al., 2002). It is well known that choline metabolism connects to the methionine (Met) cycle, as choline can be oxidised to form the methyl donor, betaine, for the conversion of homocysteine to Met, the first limiting amino acid in poultry. Several studies have suggested that dietary choline can partially replace the function of Met (Wang, 2012). Further, it bears consideration that choline is essential for the synthesis of phospholipids, such as phosphatidylcholine and membrane phospholipids, thus, preventing excessive lipid accumulation and reducing the incidence of fatty liver disease in animals (Cooke et al., 2007; Pickens et al., 2009). Previous studies have reported that the choline requirements for weight gain and feed intake of White Pekin ducks from hatch to 21 days of age are 810 and 823 mg/kg, respectively. It also suggested that higher doses of choline supplementation should be considered to completely prevent perosis and excessive lipid deposition in the liver (Wen et al., 2014). However, few studies have reported the association of choline with lipid metabolism in older breeder ducks, and the relevant information was also not provided by NRC (1994).

Additionally, there is one pathway that involves choline degradation and production of trimethylamine (TMA), which is subsequently metabolised, exclusively, into trimethylamine N‐oxide (TMAO) by hepatic flavin monooxygenase 3 (FMO3) (Shimizu et al., 2012). TMAO, a novel and independent risk factor, promotes atherosclerosis (AS) (Tang et al., 2013; Zeneng et al., 2011). Under normal FMO3 enzyme activity, dietary choline analogues can be converted to TMA in the gut, and its subsequent oxidation to form TMAO by the FMO3 enzyme can lead to excessive synthesis of TMAO in mice and chickens, resulting in the precipitation of AS in the organism under study (Koeth et al., 2013; Song et al., 2022). Ducks have lower FMO3 enzyme activity than that of other poultry (Guo, 2018); however, the effect of dietary choline on serum TMAO levels in ducks is still unknown. In addition, as an intermediate product of conversion of choline to TMAO, TMA is the main substance with fishy odour in egg yolks (Guo, 2018). Previous studies have focused on the fact that feeding ducks a high‐level choline diet (containing 4000 mg/kg choline chloride) leads to a higher egg yolk TMA level and increases the risk of fishy eggs (Guo, 2018; Honkatukia et al., 2005; Li et al., 2019). In contrast, there are few studies to assess the effect of choline on the serum lipid parameters and cardiovascular health (CVH) of older breeder ducks.

The aim of the present study was to test the efficacy of a high choline diet in improving the serum lipid parameters and to evaluate the effect of this diet on the composition of eggs of the subject animals during the late laying period. The results of the present study will increase our understanding of the function of choline in improving the health of older breeder ducks.

## 2 MATERIALS AND METHODS

### 2.1 Birds and diet

The 60 female Jingjiang ducks used in this study were obtained from the Lihu Poultry Egg Co., Ltd in the Hubei Province, China. The 60 ducks were randomly separated into six replicates of 10 birds each. Each bird was already adapted to the environment and diet. From peak production until 65 weeks of age, birds were fed a control basal diet (Table 1). All the ducks (6 replicates × 10 ducks) served as the control group until 65 weeks of age, after which, they were supplemented with 2955 mg/kg choline above and over the basal control diet, for 15 days. Accordingly, the same birds subsequently served as the choline‐supplemented group. The choline was in the form of 4000 mg/kg choline chloride with purity rated at ≥99.0% (Beijing Beshine Chemical Technology Co., Ltd, Beijing, China), similar with previous studies on chickens, quails and geese (Honkatukia et al., 2005; Mo et al., 2013; Zhong et al., 2017). The basal diet was formulated to meet standard NRC requirements (NRC, 1994), as well as the typical feeding requirements of laying ducks. The amount of supplementary choline was approximately twice the level recommended by the NRC for breeder chickens (NRC, 1994). The total choline levels of the basal diet and the choline supplemented (basal diet + 2955 mg/kg choline) were analysed using a chromatographic assay, as described by Ding and Mou (2004). The levels were found to be 1396 mg/kg and 4351 mg/kg, respectively.

**TABLE 1:** Main constituents in the basal diets of ducks

Item	Basal diet
Ingredient (%)	
Corn	60.00
Soybean meal (44%)	28.00
Calcium carbonate	10.00
Dicalcium phosphate	1.20
Premix^1^	0.80
Nutrient level^2^	
AME (kcal/kg)	2601.00
CP (%)	17.06
CF (%)	2.61
EE (%)	2.63
Ca (%)	4.35
N‐Phy‐P (%)	0.35
Lys (%)	0.89
Met (%)	0.26
Met + Cys (%)	0.53
Analysed value^3^	
Choline (mg/kg)	1396.00

^1^ Premix provided the following per kilogram of diets: Vitamin A, 8000 IU; vitamin D1, 1000 IU; vitamin E, 35 mg; vitamin B_1_, 2 mg; vitamin B_2_, 4 mg; vitamin B_6_, 2 mg; vitamin B_12_, 0.01 mg; pantothenate, 8 mg; niacin, 60 mg; folic acid, 2 mg; biotin, 1 mg; Fe, 70 mg; Cu,10 mg; Mn, 60 mg; Zn, 70 mg; Se, 0.12 mg; I, 0.48 mg; NaCl, 0.3% and choline chloride, 300 mg.

^2^ Calculated values.

^3^ Choline content in the diets was determined by ion chromatography method.

Birds were allocated to 3‐tier battery cages (cage size: 55 cm × 55 cm × 55 cm; one duck per cage) and exposed to 16 h of light/day with an intensity of 10 lx (at bird‐head level). The temperature was maintained between 22°C and 26°C throughout the experiment. The feed was offered in mash form, ad libitum, with each duck ingesting ∼150 g of feed/day. Water was supplied via nipple drinkers. All ducks remained in good health during the feeding period. No birds were culled, and none received medical intervention.

### 2.2 Sample collection and measurements

At 65 weeks of age before initiation of choline supplementation, blood samples were collected on the last day that the ducks were fed a basal diet. Subsequently, 4–6 eggs were collected from each replicate (10 birds) in the morning. On the 15th day after the choline‐supplemented diet was introduced, blood samples were also collected, and 4–6 eggs were collected from each replicate in the morning. Blood samples were collected from wing veins and then stored into a vacuum blood collection tube (without anti‐coagulant), then allowed to stand at room temperature (25°C) for 1–2 h prior to centrifugation (at 3000 × *g* for 10 min) at 4°C to obtain serum. The serum collected was stored at −20°C until analysis. On the day that the eggs were collected, the egg yolks were immediately isolated and preserved at −20°C for TMA and cholesterol analysis.

In addition, at the same time point (65 weeks and 67 weeks + 1 day), serum samples were also collected from another 60 Jingjiang ducks, who have the same genetic background and rearing conditions as above experimental flock, but without a choline‐supplemented dietary regimen. These serum samples were used to calculate the rate of change (RC) for serum physiological indices affected by age.

#### 2.2.1 Serum physiological index analysis

Serum high‐density lipoprotein cholesterol (HDL‐C), total triglyceride (TG), total cholesterol (TC), and low‐density lipoprotein cholesterol (LDL‐C) levels were determined by using commercial kits (Shanghai Kehua Bioengineering Co., Ltd., Shanghai, China). The automatic biochemical analyser used was a KHB ZY‐1280 manufactured by the Shanghai Kehua Bio‐engineering Corporation (Shanghai, China). Serum very low‐density lipoprotein (VLDL), triiodothyronine (T3), and thyroxine (T4) levels were measured by using duck VLDL, T3, and T4 enzyme‐linked immunosorbent assay kits (VLDL ELISA kit JLC20779, T3 ELISA kit JLC20684 and T4 ELISA kit JLC20829) from Shanghai Jingkang Bioengineering Co., Ltd., Shanghai, China. Other parameters were calculated as follows: TC/HDL‐C = (total cholesterol/high‐density lipoprotein cholesterol), LDL‐C/HDL‐C = (low‐density lipoprotein cholesterol/high‐density lipoprotein cholesterol), atherosclerosis index (AIS) = [(total cholesterol minus high‐density lipoprotein cholesterol)/high‐density lipoprotein cholesterol] and non‐HDL‐C = (total cholesterol minus high‐density lipoprotein cholesterol) (Cai et al., 2017). RC of serum indices within the same duck between 65 weeks and 67 weeks + 1 day was obtained by applying the following formula:

$$\mathrm{RC}\left(\%\right)=\left(X_{67\mathrm{wks}+1d}-X_{65\mathrm{wks}}\right)/{X_{65}}_{\mathrm{wks}}\times 100\%,$$

where *X*_67wks+1d_ includes the levels of serum HDL‐C, VLDL, TG, TC, LDL‐C, T3, T4, TC/HDL‐C, LDL‐C/HDL‐C, AIS and non‐HDL‐C of ducks at 67 weeks + 1 day and *X*_65wks_ includes the levels of those same indices of the same ducks at 65 weeks.

#### 2.2.2 TMA and TMAO analysis

The TMA levels in the egg yolks were measured using headspace gas chromatography as previously described (Long et al., 2017; Shimizu et al., 2012). Serum TMA and TMAO levels were also measured using headspace gas chromatography as previously described (Wang et al., 2012), with minor modifications. Serum TMAO levels were calculated from the serum total TMA level minus the serum TMA levels. A Bruker 450 gas chromatograph (Bruker Daltonics Inc., Billerica, MA, USA) equipped with a flame ionisation detector was used for these analyses. The chromatograph was also fitted with a 30 m × 0.32 mm ID‐fused silica capillary column coated with a 0.25‐μm film of DB‐Wax (J&W, Folsom, CA, USA). Operating protocols and conditions were set as follows: 40°C for 3 min, increased to 180°C at a rate of 10°C/min and maintained at 180°C for 4 min; 200°C injector temperature and 250°C detector temperature; split mode (split ratio 10:1) and hydrogen carrier gas injected at a rate of 1 ml/min.

#### 2.2.3 Egg yolk cholesterol analysis

Egg yolk cholesterol was measured using an enzymatic colorimetric method as described by Jensen et al. (2014), with minor modifications. The collected egg yolks were thoroughly mixed in a homogeniser at 4°C. Approximately 1.00 g of each egg yolk sample (accurate to 0.01 g) was mixed with 20 ml methanol in a 50 ml centrifuge tube and shaken well. Thereafter, 10 ml KOH (600 g/L) was added to each sample, and samples were kept in a water bath at 65°C for 1 h. The mixing system shook the samples once every 20–30 min until the saponification reaction was completed. Samples were then cooled to 25°C. Distilled water (20 ml) was then added, and the samples were transferred to a 125‐ml separatory funnel and extracted three times with petroleum ether (using 20, 20, and 10 ml petroleum ether each time). A sodium chloride solution (3 ml) was added to each extracted sample and the petroleum ether layer was washed with distilled water until the pH was subsequently neutralised (pH 7). The washed petroleum ether extracts were transferred to 100‐ml centrifuge tubes and concentrated by drying using rotary evaporation. Finally, the extracts were dissolved to a volume of 10 ml, with absolute ethanol, to prepare the test solutions for analysis. Absorbance was measured at a wavelength of 505 nm using an enzyme‐standardised microplate reader (SpectraMax i3x Multi‐Mode Microplate Reader; Molecular Devices, LLC, San Jose, CA, USA), and a standard curve was extrapolated. The yolk cholesterol concentrations were obtained using the standard curve.

### 2.3 Statistical analysis

The SIMCA‐P14.1 software package (Umetrics, Umea, Sweden) was applied to orthogonal partial least‐squares discriminant analysis (OPLS‐DA). Subsequently, statistical analyses were conducted using the statistical R software (version 4.0.3; The R Foundation for Statistical Computing, Vienna, Austria), and the figures were plotted using GraphPad Prism (Version 7.04; GraphPad Software, San Diego, CA, USA). Basic descriptive statistics and paired sample *t*‐tests were used to analyse the lipid parameters, as well as the TMA, TMAO, and cholesterol concentration levels between the two treatments. The correlations of the relationships of serum TMA, serum TMAO and serum physiological parameters were analysed using Spearman's correlation. *p < 0.05 and **p < 0.01 were considered statistically significant and extremely statistically significant, respectively. Data were expressed as means ± standard deviation (SD), *n* = 6 replicates × 10 ducks.

## 3 RESULTS

### 3.1 OPLS‐DA of serum samples

**FIGURE 1:**
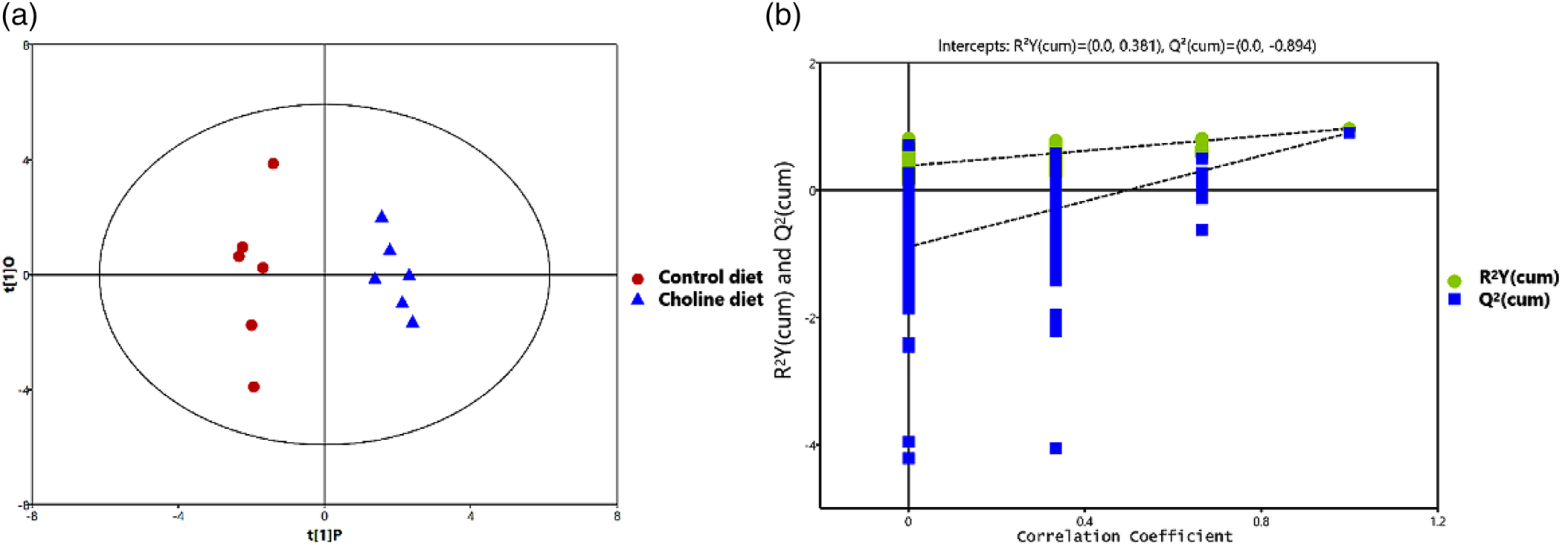
Orthogonal partial least‐squares discriminant analysis (OPLS‐DA) score plots (a) and corresponding validation plots of the permutation tests (200 times) of the OPLS‐DA models (b) for control and choline diet treatments (*n* = 6 replicates × 10 ducks)

A supervised OPLS‐DA model (Trygg et al., 2007) was constructed to investigate and analyse the separation of the control and choline diet treatments (Figure 1). As is shown in Figure 1a, the control and choline diet treatments were clearly separated. All serum samples of the score plots were within the 95% Hotelling's T‐squared ellipse. The performance scores of the OPLS‐DA model were *R* ^2^ *Y* = 0.967 and *Q* ^2^ = 0.898, which indicated that the model was stable and indicative of a robust fit and prediction. To better validate the OPLS‐DA model, a permutation test (*n* = 200) was used for verification. The corresponding results are shown in Figure 1b. The permutation test results for the *R* ^2^ *Y* and *Q* ^2^ values were 0.381 and −0.894, respectively, between the control and choline diet treatments. These results indicated that the OPLS‐DA model was not over‐fitted, showed good stability and suggested a significant metabolic change due to the choline diet.

### 3.2 Serum lipid parameters

**FIGURE 2:**
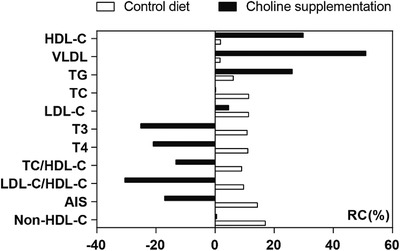
Rate of change (RC) of serum physiological indices in the same duck with or without choline supplementation. HDL‐C, high‐density lipoprotein cholesterol; VLDL, very low density lipoprotein; TG, triglyceride; TC, total cholesterol; LDL‐C, low‐density lipoprotein cholesterol; T3, triiodothyronine; T4, thyroxine; AIS = (TC − HDL‐C)/HDL‐C; non‐HDL‐C = TC − HDL‐C. RC of serum indices in the same duck between 65 weeks and 67 weeks + 1 day was obtained by applying the following formula: RC (%) = (*X*_67wks+1d_ − *X*_65wks_)/*X*_65wks_ × 100%. *X*_67wks+1d_ includes the levels of serum HDL‐C, VLDL, TG, TC, LDL‐C, T3, T4, TC/HDL‐C, LDL‐C/HDL‐C, AIS and non‐HDL‐C of ducks at 67 weeks + 1 day; and *X*_65wks_ includes the levels of those same indices of the same ducks at 65 weeks

The serum lipid parameters in the ducks from the control and choline diets are shown in Table 2. Choline dietary supplementation increased the serum HDL‐C and VLDL levels by 30% and 50% (*p* < 0.01), respectively, compared to those of the controls. Additionally, it increased the serum TG levels (*p* < 0.05) but did not change the serum TC and LDL‐C concentrations. However, it reduced the serum T3 (*p* < 0.05) and T4 (*p* < 0.01) levels in ducks. In addition, as a result of choline supplementation, the nontraditional serum lipid parameters including TC/HDL‐C, LDL‐C/HDL‐C and AIS ratios in ducks were significantly (*p* < 0.01) decreased. Of note, the AIS was decreased by 17% (*p* < 0.01) compared with the controls, although there was no significant change detected in non‐HDL‐C values in the ducks. Moreover, as is shown in Figure 2, our results also showed that the choline supplementation significantly increased the RC of HDL‐C, VLDL and TG, and reversed the RC of T3, T4, TC/HDL‐C, LDL‐C/HDL‐C and AIS compared with those in ducks on control diets. These results further implied positive effects of choline supplementation in improving the physiological indices of older ducks as measured in serum.

**TABLE 2:** Serum lipid parameters in ducks from control and choline diets

Item^1^	Control diet	Choline diet	*p* Value
Traditional lipid parameters			
HDL‐C (mmol/L)	1.14 ± 0.61	1.48 ± 0.73**	*p* < 0.01
VLDL (ng/ml)	11.00 ± 7.57	16.61 ± 7.07**	*p* < 0.01
TG (mmol/L)	13.00 ± 6.51	16.39 ± 7.87*	*p* < 0.05
TC (mmol/L)	5.20 ±2.53	5.21 ± 1.30	*p* > 0.05
LDL‐C (mmol/L)	2.62 ± 0.93	2.74 ± 1.52	*p* > 0.05
T3 (nmol/L)	3.26 ± 1.95	2.44 ± 1.23*	*p* < 0.05
T4 (ng/ml)	58.05 ± 22.83	45.91 ± 19.82**	*p* < 0.01
Nontraditional lipid parameters			
TC/HDL‐C	4.45 ± 0.81	3.86 ± 0.81 **	*p* < 0.01
LDL‐C/HDL‐C	2.62 ± 1.45	1.82 ± 0.22**	*p* < 0.01
AIS	3.45 ± 0.81	2.86 ± 0.81**	*p* < 0.01
Non‐HDL‐C (mmol/L)	3.73 ± 1.43	3.75 ± 0.64	*p* > 0.05

^1^ HDL‐C, high‐density lipoprotein cholesterol; VLDL, very low density lipoprotein; TG, triglyceride; TC, total cholesterol; LDL‐C, low‐density lipoprotein cholesterol; T3, triiodothyronine; T4, thyroxine; AIS = (TC − HDL‐C)/HDL‐C; non‐HDL‐C = TC − HDL‐C. Values are expressed as means ± SD, *n* = 6 replicates × 10 ducks.

*Significant differences at *p* < 0.05 compared to control group.

**Significant differences at *p* < 0.01 compared to control group.

### 3.3 Serum TMA and TMAO levels

**FIGURE 3:**
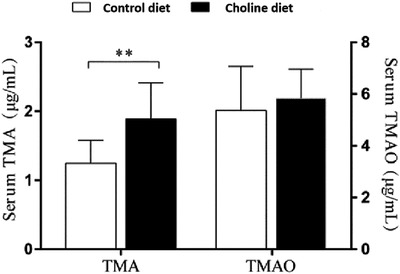
The serum TMA and TMAO levels in ducks from the control and choline diets. Bars and error bars represent means ± SD, respectively. *n* = 6 replicates × 10 ducks, **p < 0.01

The serum TMA and TMAO concentrations in the ducks from the control and choline‐supplemented diets are shown in Figure 3. Choline supplementation increased the serum TMA concentration of the ducks compared with that of when the control diet was provided (1.89 ± 0.52 μg/ml vs. 1.25 ± 0.33 μg/ml, *p* < 0.01). However, it did not change the serum TMAO concentrations of the ducks (5.37 ± 1.67 μg/ml vs. 5.85 ± 1.10 μg/ml, *p* > 0.05).

### 3.4 Egg yolk TMA and cholesterol

**FIGURE 4:**
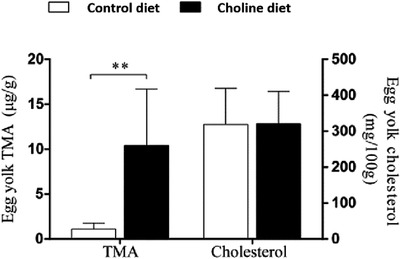
The egg yolk TMA and cholesterol levels from the control and choline diets. Bars and error bars represent means ± SD, respectively. Data represent means from 6 replicates with 4–6 eggs each, *p < 0.05, **p < 0.01

The egg yolk TMA and cholesterol concentrations from the control and choline diets are shown in Figure 4. Although choline addition increased the egg yolk TMA concentrations (10.42 ± 6.23 μg vs. 1.12 ± 0.66 μg/g, *p* < 0.01), it did not change the egg yolk cholesterol concentrations compared with the controls.

### 3.5 Correlations among the serum TMA, TMAO and lipid parameters

**FIGURE 5:**
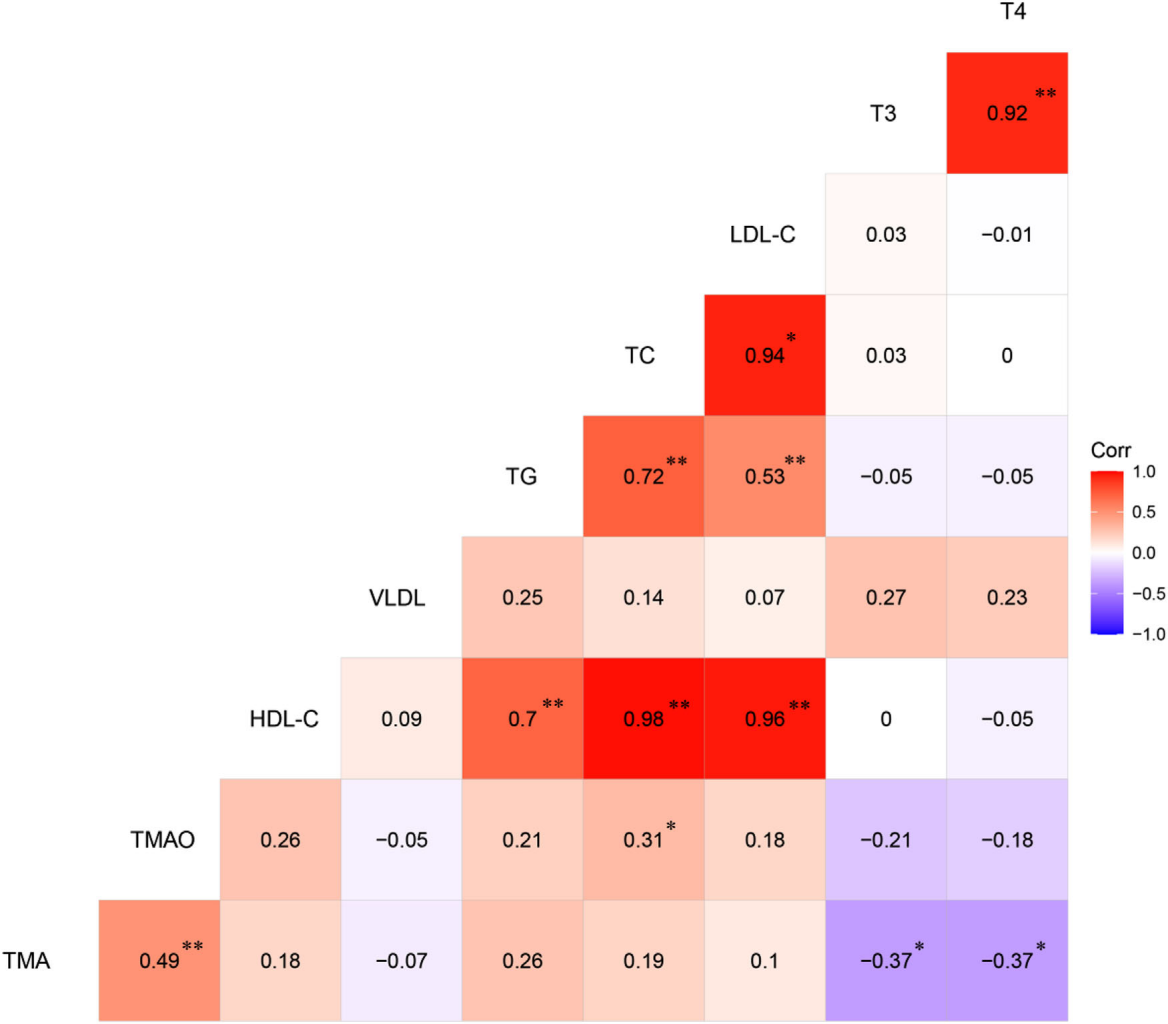
Heat map based on Spearman's correlations for the relationship of serum trimethylamine, trimethylamine N‐oxide, and physiological parameters from choline diet. The colour scale represents Spearman's correlation coefficients, with red and bluish violet representing positive and negative correlations, respectively. TMA, trimethylamine; TMAO, trimethylamine N‐oxide; HDL‐C, high‐density lipoprotein cholesterol; VLDL, very low‐density lipoprotein; TG, triglyceride; TC, total cholesterol; LDL‐C, low‐density lipoprotein cholesterol; T3, triiodothyronine; T4, thyroxine. *p < 0.05, **p < 0.01

The correlation matrix of the relationships of the serum TMA, TMAO and lipid parameters is shown in Figure 5. As an effect of choline dietary supplementation, a positive correlation between the serum TMA and TMAO levels (*r* = 0.49, *p* < 0.01), as well a positive correlation between the serum TMAO and TC levels (*r* = 0.31, *p* < 0.05) was detected in the ducks. Further, significantly (*p* < 0.01) positive correlations were also detected between serum HDL‐C, LDL‐C and TC levels when ducks were fed with the choline‐supplemented diet. However, no correlation was detected between the serum VLDL and LDL‐C levels.

## 4 DISCUSSION

Dietary choline plays important roles in lipid metabolism, and, subsequently, in the growth and reproductive performance of laying ducks (Trygg et al., 2007). Diets rich in choline (either with meat or vegetal foods) can alter the entire macrophage reverse cholesterol transport pathway (RCT) associated with lipid parameters, by impacting gut microbiota composition, as well as TMA and TMAO levels (Koeth et al., 2013; Manor et al., 2018; Zeneng et al., 2011). Lipid parameters have been well established to be closely related to CVD (Frohlich, 2003). For example, HDL‐C provides protection from AS by promoting the RCT pathway and reducing uptake of cholesterol from macrophages (Bloedon et al., 2008). Previous research has indicated that a 10 μg/ml increase in HDL‐C is associated with a 2%–4% reduction in CVD risk (Gordon et al., 1989). In our study, the choline‐supplemented diet facilitated synthesis of HDL‐C and VLDL and, thus, increased the serum HDL‐C and VLDL levels (Agnes et al., 2004). Choline can undergo phosphorylation to form phosphocholine, for synthesis of lecithin via the cytidine diphosphocholine pathway (Lien & Jan, 1999). Under the action of lecithin, free cholesterol can be converted to cholesteryl ester within the HDL‐C particle, resulting in mature HDL‐C (Lee‐Rueckert et al., 2016). In addition, it has also been suggested that the plasma HDL‐C levels correlate with the richness of gut microbiota (Tang et al., 2013). Choline diets may alter composition of the gut microbiota of ducks. Thus, these combined factors may lead to the elevation of serum HDL‐C and VLDL levels.

The observed increase of serum TG could be primarily attributed to the elevation of hepatic VLDL secretion (Walzem et al., 1999). VLDL can transport hepatic TG into the blood stream; therefore, the enhancement of hepatic VLDL secretion may protect the liver from TG accumulation (Agnes et al., 2004; Wang et al., 2016). TG, which circulates to the extra‐hepatic tissues via the circulatory system, can be catabolised into fatty acids and glycerol under the action of lipoprotein lipase and hormone‐sensitive lipase, or it is directly oxidised into carbon dioxide and water to release energy (Agnes et al., 2004; Kim et al., 2019). Furthermore, it was also detected that the choline supplemented did not change the serum LDL‐C levels of ducks, and there was no correlation between the serum VLDL and LDL‐C levels, indicating that the increased VLDL levels did not promote the synthesis of LDL‐C, a causal risk factor for AS. Further, the observed decreases of serum T3 and T4 may indicate an alleviation of hyperthyroidism of ducks in response to the choline supplementation (Mansorian et al., 2018). These results suggest that choline supplementation has a beneficial effects on the lipid metabolism of breeder ducks.

Compared with conventional lipid parameters, there is growing evidence that HDL‐C‐related ratios, such as TC/HDL‐C, LDL‐C/HDL‐C, AIS and non‐HDL‐C are superior predictors of CVD (Cai et al., 2017; Manninen et al., 1992; Zhu et al., 2015). The univariate logistic regression analyses showed that the increases of TC/HDL‐C, LDL‐C/HDL‐C and AIS significantly increased the risk of CVD (Manninen et al., 1992). In contrast, it has also been suggested that an LDL‐C/HDL‐C ratio of ≤ 2.5 indicates low risk (Manninen et al., 1992). In our study, the choline diet treatment significantly (*p* < 0.01) decreased the TC/HDL‐C, LDL‐C/HDL‐C and AIS in the ducks, illustrating that choline can improve the CVH of older ducks. These findings could be attributed to the increase in serum HDL‐C levels from choline supplementation. The positive correlation between serum HDL‐C and serum TC (*r* = 0.98, *p* < 0.01) demonstrated that HDL‐C had a profound effect on serum TC.

Choline diet could not only improve lipid parameters but also affect the TMA/TMAO/FMO3 metabolic pathway. In the present study, the choline diet treatment significantly increased the serum TMA levels of ducks, which is consistent with previous studies in laying hens (Wang et al., 2012). However, it did not affect the serum TMAO levels of the ducks. This finding is different from that of chickens fed a choline diet, which exhibit higher plasma TMAO (∼180 μg/ml) levels compared with those who were fed control diets (∼150 μg/ml) (Wang et al., 2016). Ducks generally have lower serum TMAO levels because of their low FMO3 enzyme activity (Guo, 2018). Previous studies have confirmed that TMAO levels can explain 11% of the variation in AS, and a decreased TMAO level can reduce the risk of AS in humans, mice, as well chickens (Bennett et al., 2013; Song et al., 2022). Therefore, the stability of the serum TMAO levels in ducks after choline‐rich diet introduction indicated that the choline diet did not increase the risk of CVD and might lead to an anti‐AS effect in breeder ducks, to some extent.

Our study also determined that the choline‐supplemented diet increased the egg yolk TMA levels of ducks. A region of choline can be degraded into TMA by microorganisms in the animal's intestine (Rebouche, 1991; Zhang et al., 1999; Zeisel et al., 1989), and subsequently, TMA may then be deposited in the eggs (Butler & Fenwick, 1984). TMA is the main factor leading to the fishy odour in duck eggs. An increase of TMA levels may affect the flavour of fresh eggs to a certain extent. When fresh eggs are made into reprocessed eggs, the level of TMA in the eggs is not changed (Li et al., 2019). TMA is also a common substance in many fermented foods, such as fermented skate, which has a TMA level of approximately 3 mg/g (Reynisson et al., 2012). The response to TMA also varies due to trace amine‐associated receptor 5 genotype variation in different individuals (Gisladottir et al., 2020), and people of different genders and regions have different perception abilities for eggs with a fishy odour (Li et al., 2019). At the same time, the purpose of these eggs is to hatch ducklings for the next generation, not for human consumption. Studies on chickens have shown that the egg quality is not changed by high choline levels in the diets of laying hens (Wang, 2011), and either high or low TMA levels do not affect the hatchability of chicks (Pearson & Butler, 1983). In short, our results show that dietary choline has a beneficial effect on duck lipid parameters and CVH, although it increases the TMA level in egg yolks.

Additionally, choline metabolism is also related with the Met cycle, which can provide a methyl‐group donor and facilitate the conversion of homocysteine to Met (Wang, 2012). This may imply that choline dietary supplementation could reduce the consumption of Met and improve the utilisation of protein in some low‐protein diets, which are advocated because of their low cost and utility in improving heat stress in poultry (Saleh et al., 2021). In addition, our study also found that the increasing trend of HDL‐C introduced by choline supplementation is consistent with the high‐density lipoprotein trend precipitated by a low‐protein diet (Salah, 2016). This may further imply existing correlation between choline and amino acid metabolism, dietary protein levels and changes of blood lipid parameters. These hypotheses warrant further investigation. In brief, we recommend introducing a choline supplementation into the diet of breeder ducks, to improve lipid parameters and CVH, after peak production. With the further clarification of choline function and a more precise choline requirement of ducks, we expect to obtain breeder ducks with better lipid parameters and better‐quality eggs during the late laying period.

## 5 CONCLUSIONS

Supplemental choline in the diet had a beneficial effect on lipid parameters and CVH of older breeder ducks, as it increased the serum HDL‐C levels and decreased AIS, a predictor of CVD risk, in ducks. Although it increased the egg yolk TMA levels in ducks, it did not alter the egg yolk cholesterol concentration. Our findings provide a basis for dietary choline supplementation to improve lipid parameters and CVH in older breeder ducks.

## CONFLICT OF INTEREST

We declare no financial or personal relationships with other people or organisations that might inappropriately influence our work, and we have no professional or personal interests in any product.

## ETHICS STATEMENT

The authors confirm that the ethical policies of the journal, as noted on the journal's author guidelines page, have been adhered to and the Animal Care and Use Committee of China Agricultural University (CAU20160916–2) approval has been received. The authors confirm that they have followed EU standards for the protection of animals used for scientific purposes (and feed legislation).

## AUTHOR CONTRIBUTIONS

Manuscript, data collection and formal analysis: JS. Methodology: XS. Samples collection: XL. Supervision, design, direction and funding acquisition: JZ. All authors have read and agreed to the published version of the manuscript.

## Data Availability

The data that support the findings of this study are available on the request from the corresponding author. The data are not publicly available due to privacy or ethical restrictions.
